# Ethnobotanical survey in Canhane village, district of Massingir, Mozambique: medicinal plants and traditional knowledge

**DOI:** 10.1186/1746-4269-6-33

**Published:** 2010-12-03

**Authors:** Ana Ribeiro, Maria M Romeiras, João Tavares, Maria T Faria

**Affiliations:** 1 Tropical Research Institute, Rua da Junqueira 86, 1300-344, Lisbon, Portugal; 2 Faculdade de Agronomia e Engenharia Florestal, Universidade Eduardo Mondlane, CP 257, Maputo, Mozambique

## Abstract

**Background:**

Medicinal plants are used by 80% of people from developing countries to fulfill their primary health needs, occupying a key position on plant research and medicine. Taking into account that, besides their pharmaceutical importance, these plants contribute greatly to ecosystems' stability, a continuous documentation and preservation of traditional knowledge is a priority. The objective of this study was to organize a database of medicinal plants including their applications and associated procedures in Canhane village, district of Massingir, province of Gaza, Mozambique.

**Methods:**

In order to gather information about indigenous medicinal plants and to maximize the collection of local knowledge, eleven informants were selected taking into account the dimension of the site and the fact that the vegetation presents a great homogeneity. The data were collected through intensive structured and semi-structured interviews performed during field research. Taxonomical identification of plant species was based on field observations and herbarium collections.

**Results:**

A total of 53 plant species have been reported, which were used to treat 50 different human health problems. More than half of the species were used for stomach and intestine related disturbances (including major diseases such as diarrhea and dysentery). Additionally, four species with therapeutic applications were reported for the first time, whose potential can further be exploited. The great majority of the identified species was also associated with beliefs and myths and/or used as food. In general, the community was conscientious and motivated about conservational issues and has adopted measures for the rational use of medicinal plants.

**Conclusions:**

The ethnomedicinal use of plant species was documented in the Canhane village. The local community had a rich ethnobotanical knowledge and adopted sound management conservation practices. The data compiled in this study show the social importance of the surveyed plants being a contribution to the documentation of PGR at the national and regional level.

## Background

In ancient times, medicinal plants have been used all over the world as unique sources of medicines and may constitute the most common human use of biodiversity [1,2]. According to the World Health Organization, 80% of people in developing countries still depend on local medicinal plants to fulfill their primary health needs [3]. Besides that, there is a global consensus on the benefits of phytopharmacy and at present medicinal plants occupy a key position in plant research and medicine. These facts associated with the progressive loss of traditional knowledge, due to rural exodus, and with the threats to which Plant Genetic Resources (PGR) are exposed, make the efforts to study and preserve PGR relevant in every respect. In this context, several conservation studies have been performed [4-6].

Like most African countries, Mozambique is an important repository of biological diversity. This diversity is used by ca. 90% of the country's population to fulfill its housing, food, energy and health needs. According to [7], in Mozambique approximately 15% of the total PGR (ca. 5,500 plant species) is used by rural communities for medical purposes and plays a key role in basic health care. Despite a long history of medicinal plants use in Mozambique, research on this subject is still incipient [8-10] and poorly disseminated, focusing mainly on medicinal plant markets and trade issues from Maputo province [7]. The work presented in this article reports on the utilization of medicinal plants in the Canhane village, district of Massingir, Province of Gaza. The last survey in the region dates from 1960-70 [11,12].

**Figure 1:**
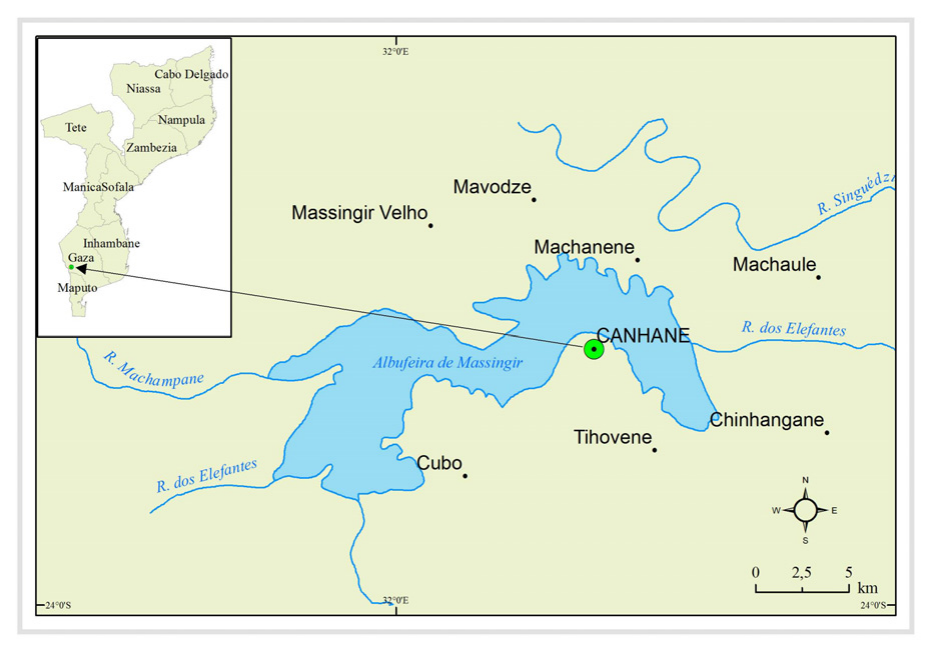
**Geographical location of the study site**. Left: Map of Mozambique illustrating the geographical position of the Province of Gaza and the District of Massingir. Right: Geographical position of Canhane within the District of Massingir.

Canhane village is located 32° 09' 30" E and 24° 4' 30" S (Figure 1). With an extension of 7,200 ha, the village has a flat landscape with slopes ranging from 0 to 2% and altitudes from 95 m N to 200 m S [13]. The climate is semi-arid with two seasons: (i) dry season (April/May to October/November), with temperatures varying from 14.5°C to 28.5°C and a maximum annual precipitation of 67.9 mm; and (ii) hot and rainy season (October/November to April/May), with temperatures ranging from 19.9°C to 32.8°C and a maximum annual precipitation of 370 mm [14]. The humidity index may vary between -50 and -70, the negative values indicating the dryness of the region [15]. The soils are essentially sandy with a low to moderate percentage of organic matter (0-3%) and thus poor for agriculture.

The village has 1357 inhabitants (51% women, 49% men) the great majority belonging to the Valoyi ("Witch doctor") family from the Changana ethnic group [16,17]. The community has poor access to water resources, health services (the closest health center is located in the Massingir village, seven Km away from Canhane), trading and communications, an obsolete energy system and an unsuccessful school system. Due to the lack of a local health center, traditional medicine plays an important role in basic health care. The main activity is agriculture, followed by livestock and fisheries. Handicraft is a tertiary activity.

The major habitat types of Canhane are woodlands, savannah and grasslands [18,19]. Currently, the vegetation communities are at different levels of degradation mainly due to human practices (e.g. production of firewood, charcoal and grazing). The over-exploitation of resources and the limiting environmental conditions seem to be associated with the decay of the resilient capacity of the ecosystems as evidenced by the occurrence of great devastated areas [17].

With this study, we intended to contribute to the conservation and valorization of the local floristic and cultural heritage. It should be noted that the study area is of particular importance, since it is located in the heart of the Limpopo National Park, which together with Kruger National Park (South Africa) and Gonarezhou National Park (Zimbabwe) constitute the Great Limpopo Transfrontier Park and Conservation Area (GLTP). The study reports on 53 medicinal plant species and their traditional applications.

## Methods

### Ethnobotanical data collection

The work was initiated with a meeting between the researchers, the community leader and the Commission for Social Management from Canhane Village, in order to: i) explain the aim and importance of the work and its integration on the Community-based Development Program; ii) get cooperation and permission to use the cultural heritage; iii) collect information for structuring the interviews; iv) give orientations for the selection of informants by age and gender; and v) plan the field activities.

Eleven informants (six men and five women) were selected as the best traditional knowledge holders. The selection criteria were based on the size of the study site, the vegetation homogeneity and on the indications provided by the community.

Due to reasons related to beliefs and myths, it was not possible to get the information directly from Witch doctors. However, it should be highlighted that most of the Canhane inhabitants belong to the Valoyi ("Witch doctor") family. Using standard methods [20,21], the data was collected through intensive structured interviews and complemented with semi-structured interviews in local language (i.e. Changana). These included: common and local name of the plant, applications, parts of the plant used, methods of preparation and administration routes. Translation to Portuguese was validated by linguistic specialists.

### Taxonomic identification

The medicinal plants reported by the informants were collected during three field surveys (in October of 2007 and in March and November of 2008). The team was accompanied by two local guides with a deep knowledge of local flora. Species identification was done during the field visits and by comparing voucher specimens with specimens deposited at the Herbarium of the Faculty of Sciences, Universidade Eduardo Mondlane (LMU, Maputo, Mozambique). The scientific names were confirmed through specialized bibliography [22-25] as well as the African Plant Database [26], Tropicos database [27] and the International Plant Names Index [28]. Additional information was gathered from the study of numerous herbarium specimens, mainly from the Tropical Research Institute Herbarium (LISC, Lisbon, Portugal).

## Results and Discussion

### Medicinal Plants' Survey

A total of 53 plant species distributed over 47 genera and 31 families were reported by the 11 informants (Table 1). All the reported species grew naturally in the area, reflecting the social importance of the local floristic resources. Most of the identified plants were shrubs or trees (15 spp. or 28.3%), herbs and trees (11 spp. for each category or 20.8%), and shrubs (nine species or 17.0%). The best represented families were Fabaceae (six species), Euphorbiaceae (four species) and Tiliaceae (three species). Altogether the 53 species were used to treat 50 different human health problems (Table 1), the great majority of which (75.5%) having more than one medical application. The most cited species were *Euclea racemosa *(ca. 82%), *Colophospermum mopane*, *Cucumis *sp. and *Elephantorrhiza elephantina *(ca. 73% each species), *Cassia abbreviata *and *Cissus quadrangularis *(ca. 64% each species), *Aloe marlothii, Maerua edulis, Secamone parvifolia *and *Terminalia sericea *(ca. 55% each species) and *Boscia albitrunca, Gossypium herbaceum *and *Gymnosporia heterophylla *(ca. 46% each species) (data not shown). The number of medicinal plants and their potential applications reflect the rich ethnomedicinal knowledge in the Canhane community. Similar potentialities were found in other African countries like Cameroon [29] and Ethiopia [30-32] as well as in non-African countries [33-35]. Certainly, there is a lot more knowledge to exploit on the topic in Mozambique.

**Table 1:** Medicinal plants (53 spp.) used in the Canhane village (2007- 2008)

Scientific name*	Local and Common name	Habit	Part used	Main Diseases	Administration Route	Method of preparation
**Acanthaceae**						

*Blepharis diversispina *(Nees) C.B. Clarke	Nchachacha wa mananga Velvet bushwillow	Sub-shrub or Herb	Fruits	Hemorrhoids	Topic	Burning and grinding
				
				Cough	Oral	Grinding and maceration
			
			Roots	Hemorrhoids	Topic	Burning and grinding
			
			Seeds	Wounds	Topic	Burning and grinding
				
				Fontanel hardening	Topic	Burning and grinding

**Aloaceae**						

*Aloe marlothii *A. Berger	Mhanga Flat-flowered aloe, Mountain aloe	Herb	Leaves (sap)	Biliary disorder; Malaria	Oral	Direct
				
				Wounds	Topic	Direct
			
			Roots; Leaves	Toothache	Oral	Direct; Decoction
				
				Liver disorder	Oral	Decoction

*Aloe zebrina *Baker	Ximhangani Small maculate aloe	Herb	Leaves (sap)	Eye treatments	Eyewash	Direct; Maceration
				
				Wounds	Topic	Direct
			
			Roots	Liver disorder	Oral	Decoction

**Amaryllidaceae**						

*Crinum stuhlmannii *Baker	Khonwua Candy-striped crinum	Herb	Stem	Swellings	Topic	Decoction

**Anacardiaceae**						

*Lannea schweinfurthii *(Engl.) Engl.	Xivombo nkanyi, xihumbunkany, munganikomo False marula	Tree	Bark	Anemia; Diarrhea; Stomach disorders	Oral	Decoction
			
			Stem	Malaria	Oral	Decoction

*Sclerocarya birrea *(A. Rich.) Hochst.	Nkanyi Marula	Tree	Bark	Anemia	Oral	Infusion
				
				Diarrhea; Stomach disorders	Oral	Scraping and decoction
				
				Hemorrhoids	Topic	Decoction and vapors; Scraping
			
			Stem	Anemia	Oral	Maceration

**Apocynaceae**						

*Sarcostemma viminale *(L.) R. Br.	Neta, netha Caustic vine	Herb (succulent)	Root	Stomach ache	Oral	Decoction
			
			Sap	Eye treatments	Eyewash	Direct

*Secamone parvifolia *(Oliv.) Bullock	Nyokani, nyoka ya yitsongo Milimili	Shrub	Roots	Deworming; Rheumatism	Oral	Decoction
				
				Epilepsy	Oral	Decoction; Heating; Grinding and water
				
				Stomach ache	Oral	Crushing and water; Decoction
			
			Stem; Roots	Varicose veins	Topic	Decoction

**Asparagaceae**						

*Asparagus africanus *Lam.	Kwangwa la tilo Bush asparagus	Shrub	Roots	Stomach disorders	Oral	Decoction
			
			Whole plant	Stomach disorders	Oral	Grinding and maceration

**Balanitaceae**						

*Balanites maughamii *Sprague	Nulu, nulo Green thorn, Y-thorned	Tree	Roots	Malaria	Oral	Scraping and infusion

**Bombacaceae**						

*Adansonia digitata *L.	Ximuwa, ximuhu, ximuvo Baobab	Tree	Bark	Debility	Bath	Maceration
			
			Roots	Diarrhea	Oral	Maceration

**Capparaceae**						

*Boscia albitrunca *(Burch.) Gilg & Gilg-Ben.	Nxunkutso, xikutse, xikutso, xikutsu, xukutsi Shepherd's tree	Shrub or tree	Leaves	Diarrhea; Hemorrhoids	Topic	Crushing and infusion

*Boscia foetida *Schinz subsp. *filipes *(Gilg) Lötter	Xicutso Bushveld shepherds tree, sandveld shepherds, tree smelly shepherds tree	Shrub	Roots	Stomach and kidney purification	Oral	Infusion

*Maerua edulis *(Gilg & Gilg-Ben. ) De Wolf	Xikolwa, xikolwe Blue-leaved bush cherry	Suffrutex or Shrub	Roots	Women fertility,	Oral	Decoction; Infusion
				
				Stomach ache	Oral	Decoction

*Maerua parvifolia *Pax	Nongonoko Dwarf bush-cherry, small-leaved maerua	Shrub	Roots	Diarrhea; Stomach ache and purification	Oral	Decoction

**Celastraceae**						

*Loeseneriella crenata *(Klotzsch) Wilczek ex N.Hallé	Lorho, nhlohlo Valley paddle-pod	Climbing shrub	Roots	Epilepsy; Stomach ache	Oral	Decoction
				
				Malnutrition	Oral; Vaccine	Burning and decoction; Grinding; Scraping and burning
			
			Stem	Antialergic	Necklace	Direct

*Gymnosporia heterophylla *(Eckl. & Zeyh.) Loes.	Xivambulani, xichangwa, libatzondze Angular-stemmed spikethorn, common spikethorn	Shrub or Small tree	Leaves	Swellings	Oral	Decoction
			
			Roots	Internal clots	Oral	Decoction
				
				Stabbing heart	Oral; Topic	Burning and grinding; Decoction; Scraping

**Combretaceae**						

*Combretum imberbe *Wawra	Mondzo Leadwood	Shrub or tree	Bark	Toothache	Oral	Decoction
			
			NA	Stomach ache	Oral	Burning and watering

*Terminalia sericea *Burch. ex DC.	Nsunsu, nkonola, kondla, mogonono Silver cluster-leaf, silver terminalia	Tree	Branches (bark)	Burns; Wounds	Topic	Drying and grinding
			
			Leaves	Stomach ache	Oral	Decoction
			
			Roots	Diarrhea	Oral	Decoction
				
				Burns; Wounds	Topic	Drying and grinding; Scraping

**Cucurbitaceae**						

*Cucumis metuliferus *E.Mey. ex Naudin	Dema	Herb	Roots	Appendicitis; Stomach ache	Oral	Decoction

*Cucumis zeyheri *Sond.	Xiyakayani, xihakahani, chihacaiane Wild cucumber	Herb	Fruits	Stomach disorders	Oral	Drying and grinding
				
				Laxative	Enema	Decanting and filtration; Grinding; Maceration
			
			Leaves	Dysentery; Laxative	Oral	Decoction; Maceration

**Dracaenaceae**						

*Sansevieria hyacinthoides *(L.) Druce	Xikwenga xa kwhati Mother-in-law tongue	Herb	Leaves	Contusions; Hemorrhoids; Rheumatism; Swellings;	Topic	Decoction and vapors; Heating
			
			Roots	Women fertility	Oral	Crushing and water
				
				Epilepsy	Oral	Decoction

**Ebenaceae**						

*Euclea racemosa *Murr.	Mulala, nhlangulo Bush guarri, glossy guarri river guarri	Shrub	Roots	Caries; Toothache	Oral	Direct (chewing)
				
				Wounds	Topic	Peeling and grinding
			
			Stem	Wounds	Bath	Cutting and water

**Euphorbiaceae**						

*Acalypha indica *L.	Ntlambissana Copperleaf, indian nettle	Herb	Leaves	Hemorrhoids	Oral; Topic	Crushing; Decoction
				
				Intestinal lavage	Enema	Grinding and decoction; Maceration
				
				Laxative	Oral; Topic	Crushing; Infusion
			
			Roots	Laxative	Oral	Decoction; Infusion
			
			Stem	Hemorrhoids	Oral	Decoction

*Androstachys johnsonii *Prain	Cimbiri Lebombo-ironwood, simbi tree	Tree	NA	NA	NA	Kept in secret by Hitch doctors

*Flueggea virosa *(Roxb. ex Willd.) Voigt	Nsangasi, sangasi Snowberry tree, whiteberry bush	Shrub	Branches	Abcesses	Topic	Heating (with castor oil on top)

*Spirostachys africana *Sond.	Xilangamahlo, dzanvori African Sandal, tamboti	Tree	Bark	Debility (HIV-AIDS)	Oral	Decoction (in milk)
			
			Sap	Ear and eye treatments	Topic	Direct
			
			Stem	Burns, Wounds	Topic	Burning; Grinding; Scraping

**Fabaceae**						

*Cassia abbreviata *Oliv.	Lumanyama Longtail cassia, sjambok pod	Tree	Fruits	Eye treatments	Eyewash	Heating and grinding
			
			Leaves, roots and stems (mix)	Stomach ache	Oral	Infusion
			
			Roots (bark)	Diarrhea	Oral	Decoction
			
			Stem	Malaria; Stomach ache	Oral	Decoction

*Colophospermum mopane *(Benth.) Léonard	Gungwa, nxanati, nxanatsi, mesanya Mopane	Shrub or Tree	Bark	Bleeding; Dysentery; Stomach ache	Oral	Decoction
			
			Leaves	Stomach ache	Oral	Crushing; Direct (chewing); Infusion
				
				Dysentery	Oral	Grinding and water
			
			Roots	Stomach ache	Oral	Decoction
			
			Stem; Stem and leaves (mix)	Stomach ache; Diarrhea	Oral	Decoction; Infusion

*Dalbergia melanoxylon *Guill. & Perr.	Xipaladze, xiphalanzi African blackwood	Shrub or Tree	Roots	Toothache	Oral	Decoction

*Dichrostachys cinerea *(L.) Wight & Arn	Ndzenga, ntsenga, ndzhenga Small-leaved sickle bush	Shrub or Small tree	Roots	Skeletal disorders	Topic; Vaccine	Burning and grinding; Scraping
			
			Roots (sap)	Laxative	Oral	Direct

*Elephantorrhiza elephantina *(Burch.) Skeels	Xivurayi Dwarf elephant's root	Shrub or Sub-shrub	Roots	Anemia	Oral	Cutting and maceration; Decoction and grinding
				
				Pain killer, Fever	Oral	Decoction

*Guibourtia conjugata *(Bolle) J. Léonard	Ntsotso Small copalwood, small false mopane	Tree	Leaves	Stomach disorders	Oral	Crushing and water
			
			Roots	Intense cough	Oral	Decoction

**Malvaceae**						

*Gossypium herbaceum *L.	Thonji ra khwati, thondji la khwati, nuba Wild cotton	Sub-shrub	Fruits	Ear treatment	Topic	Direct
			
			Roots	Vomits control	Oral	Decoction
				
				Tonic	Oral	Decoction

*Hibiscus meyeri *Harv.	Muxaxayevu, kongowa, kloklonya, muchachanyevo Dainty white wild hibiscus, lebombo hibiscus	Herb	Roots	Tonic, Stabbing heart	Oral	Decoction

**Meliaceae**						

*Trichilia emetica *Vahl subsp. *emetica*	Nkuhlu Natal-mahogany	Tree	Branches (sap)	Stomach ache	Oral	Direct (chewing)
			
			Roots	Contraceptive	Oral	Infusion

**Menispermaceae**						

*Tinospora caffra *(Miers) Troupin	Nyokani ya yikulo, nyoka ya yikulu Orange grape creeper	Creeper	Leaves	Paralysis and Children diseases	Oral	Decoction
			
			Roots	Epilepsy; Pain killer;; Paralysis and Children diseases; Stomach ache	Oral	Decoction; Infusion
			
			Stem	Epilepsy	Oral	Decoction

**Moraceae**						

*Ficus sycomorus *L.	Nkuwa Common cluster fig, sycamore fig	Tree	Sap	Ringworm	Topic	Direct

**Olacaceae**						

*Olax dissitiflora *Oliv.	Nkondzomhuntana, ximanimurhi, nondzomuntana Small sourplum, small-fruit olax	Shrub or Tree	Leaves	Wounds	Topic	Grinding

*Ximenia americana *L.	Ntsengele, matsengele, tsingela Blue sourplum, small sourplum	Shrub or Tree	Roots	Antiabortifacients, HIV-AIDS, Menstrual cycle, Stabbing heart, Stomach ache, Women fertility,	Oral	Decoction
				
				Wounds	Topic	Drying and grinding

**Orchidaceae**						

*Ansellia africana *Lindl.	Phakama Leopard orchid, monkey sugarcane, mopane orchid, tree orchid	Herb (Epiphyte)	Fruits	Cough, Rheumatism	Necklace, Bath	Direct; Heating and grinding
			
			Fruits and stem (mix)	Cough	Oral	Decoction

**Poaceae**						

*Cynodon dactylon *(L.) Pers.	Rintlhangi, nulangi-rithangi Bermuda grass	Herb	Leaves	Antiabortifacients	Oral	Grinding and water

**Ptaeroxylaceae**						

*Ptaeroxylon obliquum *Radlk.	Ndzharhi Sneezewood	Shrub or Tree	Sap	Stomach ache	Oral	Direct

**Rubiaceae**						

*Gardenia volkensii *K. Schum.	Xitsalala Bushveld, savanna or woodland gardenia, transvaal gardenia	Shrub or Tree	Leaves	Stomach ache	Oral	Grinding and water

**Rutaceae**						

*Zanthoxylum humile *(E.A. Bruce) P. G. Waterman	Manungwani, manongwane, manungwame Hairy knobwood	Shrub	Roots	Mouth anesthetic; Toothache	Oral; Topic	Decoction; Peeling, grinding, drying. and grinding
				
				Wounds and Burns	Topic	Peeling, grinding, drying. and grinding
			
			Stem	Pain killer	Vaccine	Burning

**Sapotaceae**						

*Manilkara mochisia *(Baker) Dubard	N'whamba, wambo, n'wambu Lowveld milkberry	Shrub or tree	Roots	Toothache	Oral; Topic	Decoction; Maceration and scrapping
				
				Ear treatments	Topic	Direct

**Strychnaceae**						

*Strychnos madagascariensis *Spreng. ex Baker	Nkwankwa Black monkey-orange, hairy-leaved monkey-orange	Shrub or Tree	Roots	Fever	Oral	Peeling and decoction

**Sterculiaceae**						

*Hermannia micropetala *Harv. & Sond.	Sindzambita, xisindzambita Cactus wine, wild grape	Shrub or Sub-shrub	Fruits	Laxative	Topic	Juice
			
			Leaves	Laxative	Topic	Juice
			
			Roots	Fontanel hardening	Topic	Burning and oil

**Tiliaceae**						

*Grewia flavescens *Juss. *var. flavescens*	Nsihana, nsiphane, dzuwa wa mananga Donkeyberry, Sandpaper raisin, Rough-leaved raisin	Climbing shrub	Leaves	Stomach disorders	NA	NA

*Grewia hexamita *Burret	Nsihana, nsihani, nsihane, nsiphane Giant grewia, Giant raisin	Shrub or Tree	Roots	Menstrual cycle, Women Fertility	Oral	Infusion
			
			Sap	Post-delivery cleaning	Oral	Direct

*Grewia monticola *Sond.	Nsihana, nsihani, nsiphane Grey grewia, Grey raisin, Silver raisin	Shrub or Tree	Fruits	Ear treatments	Topic	Heating and grinding
			
			Fruits; Seeds	Wounds	Topic	Heating and grinding
			
			Roots	Diarrhea	Oral	Decoction
			
			Stem	Swellings	Topic	Heating

**Vitaceae**						

*Cissus cornifolia *(Bak.) Planch.	Mphesani, mphensana Ivy-grape	Shrub or Tree	Roots	Burns; Wounds	Topic	Crushing

*Cissus quadrangularis *L.	Covoloti, Covoluti Cactus vine, wild grape	Creeper (succulent)	Roots	Ear treatments	Topic	Warming and squeezing
			
			Sap	Ear treatments; Wounds	Topic	Direct
			
			Seeds	Antidote; Wounds	Topic	Grinding
				
			Stem	Sprains; Swellings	Topic	Heating
				
				Cough	Oral	Cutting and decoction
				Antidote; Wounds	Topic	Crushing and water

Family, scientific, local and common names, growth habit, parts used, main diseases, administration route and method of preparation.

*Scientific names are according to [22-28]

NA = Not Available

More than half of the reported species (54.7%) were used for stomach and intestine related disturbances (Table 2). Of these, almost 38% were used to treat diarrhea and dysentery, a major concern in the region. In fact, in Mozambique diarrhea has for a long time been associated with a complex array of illnesses. Amongst them, dysentery and cholera usually have a high mortality rate if not treated promptly [10]. The use of traditional medicinal plants seems to play a major role in controlling diarrhea-associated diseases.

**Table 2:** Distribution by category of disease

Analgesic, anti-inflammatory and antipyretic	*Adansonia digitata*, *Cissus quadrangularis*, *Crinum stuhlmannii*, *Dichrostachys cinerea, Elephantorrhiza elephantina*, *Grewia monticola*, *Gymnosporia heterophylla*, *Sansevieria hyacinthoides*, *Strychnos madagascariensis, Tinospora caffra*, *Zanthoxylum humile*
Anemia	*Elephantorrhiza elephantina, Lannea schweinfurthii, Sclerocarya birrea*

Antialergic	*Loeseneriella crenata*

Antidote	*Cissus quadrangularis*

Appendicitis	*Cucumis metuliflerus*

Bleeding	*Colophospermum mopane*

Burns	*Cissus cornifolia*, *Spirostachys africana*, *Terminalia sericea*, *Zanthoxylum humile*

Cough	*Ansellia africana*, *Blepharis diversispina*, *Cissus quadrangularis, Guibourtia conjugata*

Debility and malnutrition	*Gossypium herbaceum*, *Hibiscus meyeri*, *Loeseneriella crenata*, *Spirostachys africana*

Dentistry	*Aloe marlothii*, *Boscia albitrunca*, *Combretum imberbe*, *Dalbergia melanoxylon*, *Euclea racemosa*, *Flueggea virosa, Manilkara mochisia*, *Zanthoxylum humile*

Deworming	*Secamone parvifolia*

Ear diseases	*Cissus quadrangularis*, *Gossypium herbaceum*, *Grewia monticola*, *Manilkara mochisia, Spirostachys africana*

Epilepsy	*Loeseneriella crenata*, *Sansevieria hyacinthoides*, *Secamone parvifolia*, *Tinospora caffra*

Eye diseases	*Aloe zebrina*, *Cassia abbreviata*, *Sarcostemma viminale, Spirostachys africana*

Ginecology	*Cynodon dactylon, Grewia hexamita*, *Maerua edulis*, *Sansevieria hyacinthoides, Trichilia emetica, Ximenia americana*

Heart	*Gymnosporia heterophylla*, *Hibiscus meyeri*, *Ximenia americana*

Hemorrhoids	*Acalypha indica*, *Blepharis diversispina*, *Boscia albitrunca*, *Sansevieria hyacinthoides, Sclerocarya birrea*

HIV-SIDA	*Ximenia americana*

Internal clots	*Gymnosporia heterophylla*

Kidney disorders	*Boscia foetida*

Liver disorders	*Aloe marlothii, Aloe zebrina*

Malaria	*Aloe marlothii*, *Balanites maughamii*, *Cassia abbreviata*, *Lannea schweinfurthii*

Non-identified diseases	*Androstachys johnsonii*

Paralysis and other children diseases	*Tinospora caffra*

Ringworm	*Ficus sycomorus*

Skeletal structure	*Dichrostachys cinerea*

Stomach and intestine disorders	*Acalypha indica*, *Adansonia digitata*, *Asparagus africanus*, *Boscia albitrunca*, *Boscia foetida*, *Cassia abbreviata*, *Colophospermum mopane*, *Combretum imberbe*, *Cucumis metuliflerus*, *Cucumis zeyheri*, *Dichrostachys cinerea*, *Hermannia micropetala, Gardenia volkensii*, *Grewia flavescens*, *Grewia monticola*, *Guibourtia conjugata*, *Gossypium herbaceum, Lannea schweinfurthii*, *Loeseneriella crenata*, *Maerua edulis*, *Maerua parvifolia*, *Ptaeroxylon obliquum*, *Sarcostemma viminale*, *Secamone parvifolia*, *Sclerocarya birrea*, *Terminalia sericea*, *Tinospora caffra*, *Trichilia emetica*, *Ximenia americana*

Varicose veins	*Secamone parvifolia*

Wounds	*Aloe marlothii*, *Aloe zebrina*, *Blepharis diversispina*, *Cissus cornifolia*, *Cissus quadrangularis*, *Euclea racemosa*, *Grewia monticola*, *Olax dissitiflora*, *Spirostachys africana*, *Terminalia sericea*, *Ximenia americana*, *Zanthoxylum humile*

Distribution of the 53 medicinal plant species within different disease categories (Canhane, 2007-2008).

Around 23% of the surveyd species were used as analgesic, anti-inflammatory or anti-pyretic and for wound treatment, 15% for dentistry and 11% for gynecology-related problems. Approximately 9% of the reported species were used to treat ear diseases and hemorrhoids, 8% for burns, cough, debility and malnutrition, epilepsy, eye diseases and malaria, and 6% for heart problems. Only one species, *Ximenia americana *(ca. 2%) was used against HIV-AIDS. Thus, looking at the three major national health concerns, namely diarrhea and dysentery, malaria and HIV-AIDS a considerable number of potentialities are available for the first group (11 species), while moderate (four species) and low (one species) alternatives can be exploited for malaria and HIV-AIDS. In fact, several pharmacological studies of these three groups of human ailments are available for most of the species reported in the present survey [36-42].

With the exception of six species (*Blepharis diversispina, Grewia flavescens, Guibourtia conjugata, Hermannia micropetala, Loeseneriella crenata, Zanthoxylum humile*), all species under study have been reported as medicinal plants in other African countries [11,43-46]. While the use of *G. flavescens *and *Z. humile *by traditional healers has been reported in India and Mozambique, respectively [7,47], as far as our literature review goes, four species (i.e. *B. diversispina, G. conjugata, H. micropetala*, and *L. crenata*) were reported here for the first time. Of these, only two genera have been associated with ethonomedicine: the genus *Blepharis *[48] and the genus *Loeseneriella *(*L. obtusifolia*). Thus, these species constitute new potential sources of natural medicines.

From the 53 species, nine were reported previously by [10] and 3 by [7] in studies conducted in the province of Maputo. Besides that, several other species belonging to 11 genera (*Aloe, Asparagus, Boscia, Cissus, Crinum, Cucumis, Ficus, Grewia, Maerua, Secamone, Strychnos*) were also reported as medicinal species [7]. The potential medicinal plant markets from the southern provinces of Maputo and Gaza seem to be different. This may reflect the rich ethonomedicinal potential which exists in the entire country.

A comparative analysis with local specific ethnobotanical literature [11,12,46] and complementary information gathered from the LISC Herbarium plant collections, identified 25 different plant species used for medicinal purposes (Table 3) of which only two, *Combretum imberbe *and *Lannea schweinfurthii*, are common to those reported in this study. Regarding their applications, similarities were found for *C. imberbe *(stomach disorders) and *L. schweinfurthii *(diarrhea and stomach disorders). According to the available data, *C. imberbe *was also used to treat schistosomiasis and *L. schweinfurthii *to treat tuberculosis, while in our survey they were additionally indicated for the treatment of toothache (*C. imberbe*), anemia and malaria (*L. schweinfurthii*). Because the older surveys did not specifically target medicinal plants, we believe that our data are more accurate in what concerns the applications of these two species. This fact may also explain why the great majority of the species reported 40 years ago (23 out of 25 or 92%) does not overlap with those identified in this survey. However, the possibility of loss of genetic resources and/or traditional knowledge should also be considered.

**Table 3:** List of medicinal plants (25 spp.) surveyed in the district of Massingir in 1960-70.

Scientific name*	Local name	Diseases- Gaza district
**Alismataceae**		

*Limnophyton obtusifolium *(L.) Miq.	NA	Ear diseases

**Amaranthaceae**		

*Chenopodium ambrosioides *L.	Kanunka uncono	Intestinal ulcers; Stomach-aches

*Hermbstaedtia odorata *(Burch.) T. Cooke	Chomeli	Diuretics; Stomach wash

**Anacardiaceae**		

*Lannea schweinfurthii *(Engl.) Engl.	chiumbocanhe, chebombocanho, munganicomo	Abdominal pain; Choleric diarrhea; Cough; Tuberculosis

*Ozoroa obovata *(Oliv.) R.Fern. & A. Fern.	xinungu, chimungumango, chinungo, chinungumafe, chinungumafi	Diarrhea; Laxative; Pain

*Sclerocarya birrea *Sond.	canhi (tree and fruit), tsula (tree), ditsula (fruit)	Diarrhea; dysentery

**Apocynaceae**		

*Adenium multiflorum *Klotzsch	chimua	Male sterility; Sexual performance

*Pergularia daemia *(Forssk.) Chiov.	furana	Antiemetic; Cough

**Araceae**		

*Stylochiton natalensis *Schott	NA	Ear diseases; Respiratory diseases; Tranquilizing

**Asteraceae**		

*Ageratum conyzoides *L.	NA	Abdominal disorders; Laxative

**Burseraceae**		

*Commiphora africana *(A. Rich.) Engl.	NA	Abdominal disorders; Asthma; Head ache; Stomach ache

**Capparaceae**		

*Boscia mossambicensis *Klotzsch	Chimapamapane, chicutlu	Eye disinfectant

*Cadaba natalensis *Sond.	tssatssassana, mejacocone	Tuberculosis

*Capparis tomentosa *Lam.	caua, cahu	Respiratory diseases; Tuberculosis

*Cladostemon kirkii *(Oliv.) Pax & Gilg	tumangoma, mahuco, maúco, buguane, tambocolata	Abdominal disorders; Colds; Sexual performance; Venereal diseases

*Thilachium africanum *Lour.	compfa, compha	Asthma; Diarrhoea; Edema; General pain; Vomiting

**Celastraceae**		

*Elaeodendron schlechteranum *(Loes.) Loes.	chigugutzo; chigugusse	Aphrodisiac; Deworming.

*Maytenus senegalensis *(Lam.) Exell	Chixangua, Chichangua; chilhangua	Bilharziosis; Bronchitis and tuberculosis; Convulsions; Diarrhoea and dysentery; Male and female sterility

**Combretaceae**		

*Combretum apiculatum *Sond.	Chivonzôane, samabulile	Abdominal pain; Conjunctivitis

*Combretum imberbe *Wawra	Monzou; mondzo	Bilharziosis; Stomach-aches

*Combretum microphyllum *Klotzsch	Funté, mumbambanguene pfunte	Abdominal pain; Bilharziosis; Diarrhoea; Female sterility

*Combretum molle *R.Br ex G. Don	Chicucudze, xicucutce	Antiabortifacients, Dysentery

*Combretum mossambicense *(Klotzsch) Engl.	Futé, funté, fute	Diarrhoea; Laxative

*Combretum zeyheri *Sond.	NA	Eye cleaning

*Pteleopsis myrtifolia *(M.A. Lawson) Engl. & Diels	Ludzane	Fever; Madness Male and female sterility

NA = Not Available

The table provides the family, scientific and local names, habit and diseases of 25 spp. surveyed in the district of Massingir mainly during the 60-70 s. *[11,12,43]

**Figure 2:**
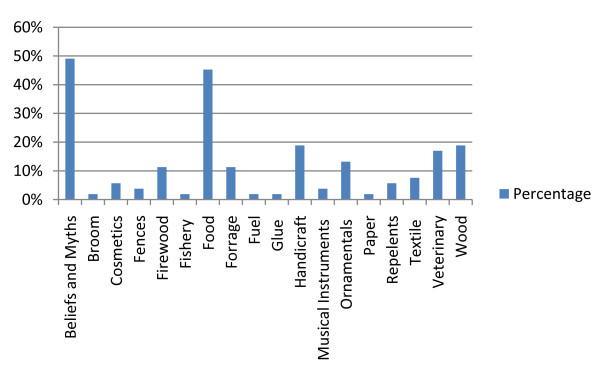
**Non-medical applications**. Non-medical applications of the 53 medicinal plant species (Canhane, 2007- 2008).

The great majority of the identified species (46 spp. or 86.8%) were also used for other purposes than medicine (Table 4; Figure 2). The major groups of applications were associated with beliefs and myths (26 spp. or ca. 49%) or used as food (24 spp. or ca. 45%). Wood production, handicraft and veterinary were the third major class of application, with 10 (ca. 19%), 9 (ca. 17%) and 8 (ca. 15%) species, respectively. This reinforces the socio-economic importance of the reported species, placing them in a privileged position for conservational aspects and income-generating purposes.

**Table 4:** Other applications of the surveyed plant species from Canhane village (2007- 2008).

Scientific name	Other Applications
**Acanthaceae**	

*Blepharis diversispina *(Nees) C.B. Clarke	Beliefs and myths

**Aloaceae**	

*Aloe marlothii *A. Berger	Beliefs and myths; Food (nectar); Veterinary (cattle diseases)

*Aloe zebrina *Baker	Food (leaves); Veterinary (cattle, chicken and lamb's diseases)

**Anacardiaceae**	

*Lannea schweinfurthii *(Engl.) Engl.	Beliefs and myths; Food (fruits); Handicraft, Ornamental

*Sclerocarya birrea *Hochst.	Beliefs and myths; Food (fruits); Handicraft; Ornamental; Wood

**Apocynaceae**	

*Sarcostemma viminale *(L.) R.Br.	Food (fruits); Forage

**Asparagaceae**	

*Asparagus africanus *Lam.	Beliefs and myths

**Balanitaceae**	

*Balanites maughamii *Sprague	Beliefs and myths; Forage; Ornamental; Wood

**Bombacaceae**	

*Adansonia digitata *L.	Food (fruits); Paper

**Capparaceae**	

*Boscia albitrunca *(Burch.) Gilg & Gilg-Ben.	Food (fruits); Firewood; Handicraft; Wood

*Maerua edulis *(Gilg & Gilg-Ben.) DeWolf	Food (Fruits)

*Maerua parvifolia *Pax	Beliefs and myths; Fishery; Food (Fruits)

**Celastraceae**	

*Loeseneriella crenata *(Klotzsch) Wilczek ex N. Hallé	Beliefs and myths; Carts and animal traction; Textile

**Combretaceae**	

*Combretum imberbe *Wawra	Charcoal; Firewood; Kitchen handicraft; Wood

*Terminalia sericea *Burch. ex DC.	Beliefs and myths; Fiber; Firewood; Handicraft; Ornamental; Wood

**Cucurbitaceae**	

*Cucumis zeyheri *Sond.	Beliefs and myths; Food (leaves); Veterinary (Cattle and lambs wounds)

*Cucumis metuliferus *E.Mey. ex Naudin	Veterinary (Goat diseases, Newcastle disease)

**Dracaenaceae**	

*Sansevieria hyacinthoides *(L.) Druce	Beliefs and myths; Textile

**Ebenaceae**	

*Euclea racemosa *Murr.	Cosmetics; Food (fruits)

**Euphorbiaceae**	

*Androstachys johnsonii *Prain	Wood

*Flueggea virosa *(Roxb. ex Willd.) Voigt	Beliefs and myths; Food (fruits)

*Spirostachys africana *Sond.	Veterinary (cattle eye diseases); Wood

**Fabaceae**	

*Cassia abbreviata *Oliv.	Beliefs and myths; Ornamental; Wood

*Colophospermum mopane *(Benth.) Léonard	Charcoal; Firewood; Handicraft; Wood

*Dalbergia melanoxylon *Guill. & Perr	Handicraft; Musical instruments

*Dichrostachys cinerea *(L.) Wight & Arn	Beliefs and myths; Farm fencing; Forage

*Elephantorrhiza elephantina *(Burch.) Skeels	Beliefs and myths

*Guibourtia conjugata *(Bolle) J.Léonard	Beliefs and myths; Firewood; Glue; Handicraft; Ornamental

**Malvaceae**	

*Gossypium herbaceum *L.	Food (Leaves); Textile

*Hibiscus meyeri *Harv.	Aphrodisiac; Broom

**Meliaceae**	

*Trichilia emetica *Vahl subsp. *emetica*	Cosmetics; Food (fruits, seeds)

**Moraceae**	

*Ficus sycomorus *L.	Beliefs and myths; Food (fruits); Forage

**Olacaceae**	

*Olax dissitiflora *Oliv.	Beliefs and myths; Food (fruits); Forage; Wood

*Ximenia americana *L.	Beliefs and myths; Cosmetics; Farm fencing; Food (fruits, seeds); Veterinary (cattle wounds)

**Orchidaceae**	

*Ansellia africana *Lindl.	Beliefs and myths

**Poaceae**	

*Cynodon dactylon *(L.) Pers.	Forage

**Rubiaceae**	

*Gardenia volkensii *K. Schum.	Beliefs and myths; Food (fruits); Textile

**Rutaceae**	

*Zanthoxylum humile *(E.A. Bruce) P.G. Waterman	Beliefs and myths; Snake repellent

**Sapotaceae**	

*Manilkara mochisia (Baker) *Dubard	Food (fruits)

**Strychnaceae**	

*Strychnos madagascariensis *Spreng. ex Baker	Beliefs and myths; Food (fruits); Handicrafts; Musical instruments

**Sterculiaceae**	

*Hermannia micropetala *Harv. & Sond.	Beliefs and myths

**Tiliaceae**	

*Grewia flavescens *Juss. *var. flavescens*	Beliefs and myths; Food (fruits)

*Grewia hexamita *Burret	Food (fruits); Handicraft

*Grewia monticola *Sond.	Food (fruits); Ornamental; Veterinary (relieves cow's pain during calf-birth)

**Vitaceae**	

*Cissus cornifolia *(Bak.) Planch.	Food (fruits); Repellent; Veterinary (cattle wounds)

*Cissus quadrangularis *L.	Beliefs and myths; Repellent; Veterinary (cattle Newcastle disease, wounds)

The table presents a list of 46 spp. which, besides their medicinal use, are used for non-medical purposes (e.g. applications related to beliefs and myths, food, handicraft, animal diseases, ornamental).

### Plant parts used, methods of preparation and administration routes

**Figure 3:**
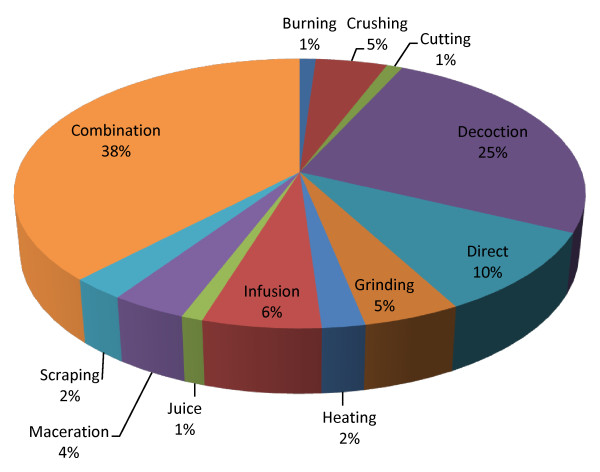
**Methods of preparation**. Methods of the 53 medicinal plant species (Canhane, 2007-2008).

**Figure 4:**
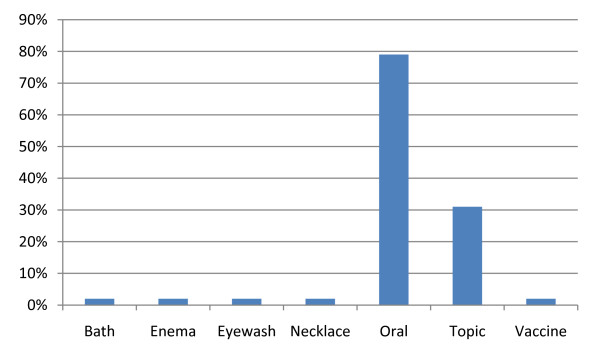
**Administration routes**. Administration routes of the 53 medicinal plant species (Canhane, 2007-2008).

Several plant parts were used (Table 1), the most frequent being roots (38.8%), followed by leaves (17.5%), stems (13.6%), fruits (8.8%), bark (5.8%), sap (5.8%), combinations of plant organs (3.9%), branches (2.9%) and seeds (2.9%). Regarding the methods of preparation (Figure 3), in many cases (38%) a combination of methods was used. The most common method was decoction (25%), followed by direct consumption (10%), infusion (6%), crushing (5%), grinding (5%), maceration (4%), scraping (2%), heating (2%), burning (1%), cutting (1%) and juice (1%). Fifty nine percent of the medicines were administered orally, 31% topically and only 10% through vaccine, bath, enema, eyewash and necklace (ca. 2% for each mode) (Figure 4). In general, the results seem to follow the pattern of medicinal plant uses in Africa [26,28,49] except that in Canhane, instead of leaves, roots occupy the top position which is concordant with the results from [7]. Consistent with the findings of [28,49] in Kenya and Ethiopia respectively, is the lack of standardized dosage and quality control.

### Conservational aspects

In general, the community was conscientious and motivated regarding conservational issues and had adopted sound measures for the rational use of medicinal plants. Conservation in farms or home gardens was performed for the most commonly used plants, namely *Aloe marlothii, A. zebrina, B. albitrunca, C. mopane, C. zeyheri, E. racemosa, Ficus sycomorus, Flueggea virosa, Grewia hexamita*, *G. monticola*, *H. micropetala, Sclerocarya birrea *and *T. sericea*. Additionally, the intensity and frequency of exploitation was controlled and there were local rules to protect native plant species, particularly *Adansonia digitata*, *B. discolor*, *Cissus cornifolia*, *C. mopane*, *E. elephantina*, *F. sycomorus*, *F. virosa*, *G. monticola*, *G. conjugata*, *Manilkara mochisia*, *S. birrea*, and *Strychnos madagascariensis*. Other conservation measures included community guards in protected places to control fires and logging, mostly due to South African migrants. On the other hand, trading was controlled and confined to the village.

## Conclusions

This study shows the social importance of the floristic richness in the Canhane village, particularly regarding the significance of medicinal plants in primary healthcare. This is reflected in the great diversity of plants used for medical purposes as well as in the wide range of their applications and associated procedures. The data compiled in this study are a contribution to the documentation of PGR at the national and regional level and can serve as a basis to develop larger and interdisciplinary studies.

## List of abbreviations

GLTP: Great Limpopo Transfrontier Park and Conservation Area; PGR: Plant Genetic Resources.

## Competing interests

The authors declare that they have no competing interests.

## Authors' contributions

The design, planning, field survey and taxonomic analysis was coordinated and conducted by TF. AR and TF performed the data processing and analysis. The taxonomic revision was done by MMR and JT. Data from other geographical regions and from 1960-70 was retrieved by MMR, JT and TF. Literature retrieval was done by AR and MMR. AR wrote the manuscript, which was revised by MMR and TF. All authors read and approved the manuscript.

## References

[B1] Hamilton AC Medicinal plants, conservation and livelihoods Biodiver Conserv 2004 13 1477 1517 10.1023/B:BIOC.0000021333.23413.42

[B2] Hiremath VT Taranath TC Traditional phytotherapy for snake bites by tribes of Chitradurga District, Karnataka, India Ethnobot Leaflets 2010 14 120 25

[B3] World Health Organization WHO traditional medicine strategy 2002-2005 2002 World Health Organization

[B4] de Vicente MC Guzmán FA Engels J Rao VA Ruane J, Sonnino A Genetic characterization and its use in decision-making for the conservation of crop germplasm The role of biotechnology in exploring and protecting agricultural genetic resources 2006 FAO of the United Nations 2129 138

[B5] Fraleigh B Ruane J, Sonnino A Global overview of crop genetic resources The role of biotechnology in exploring and protecting agricultural genetic resources 2006 FAO of the United Nations 21 32

[B6] Gepts P Plant genetic resources conservation and utilization: the accomplishments and future of a societal insurance policy Crop Sci 2006 46 2278 2292 10.2135/cropsci2006.03.0169gas

[B7] Krog M Falcão MP Olsen CS Medicinal plant markets and trade in Maputo, Mozambique Forest & Landscape Working Papers 16, Danish Center for forest landscaping and planning, KVL 2006

[B8] Jansen PCM Mendes O Plantas medicinais - Seu uso traditional em Moçambique Tomo 3. Ministério da Saúde, Maputo 1990

[B9] Chamba E Mangue P The role of traditional structures in conservation of natural resources: Potone case study3rd Regional Workshop of CASS/PLASS Community based natural resource management programme, Maputo 2001

[B10] Bandeira SO Gaspar F Pagula FP African ethnobotany and healthcare: Emphasis on Mozambique Pharm Biol 2001 39 70 73 10.1076/phbi.39.7.70.5873 21554173

[B11] Watt JM Breyer-Brandwijk MG Medicinal and poisonous plants of Southern and Eastern Africa 1962 Edinburg and London: E & S Livingstone Ltd

[B12] Faria MT Revisão de algumas Combretaceae de Moçambique Mem Inst Inv Agron Moç 1973 4 37 45

[B13] Sitoe A Chaúque A Plano de Gestão dos Recursos Naturais da Comunidade de Canhane, Distrito de Massingir Relatório de Consultoria. Lupa, Associação para o Desenvolvimento da Comunidade 2008

[B14] Ministério da Administração Estatal O perfil do distrito de Massingir Série: Perfis distritais 2005

[B15] Reddy SJ Agroclimate of Mozambique as relevant to dry-land agriculture Instituto de Investigação Agronómica de Moçambique 2008

[B16] HELVETAS, Moçambique Projecto de Desenvolvimento de Turismo baseado na Comunidade de Canhane, Distrito de Massingir-Gaza (Área de Conservação Transfronteira do Grande Limpopo) 2002

[B17] Faria T Etnobotânica da aldeia de Canhane. Plantas úteis, saberes locais e tradições 2010 Maputo: Diname Pub in press

[B18] Gomes PJ Barbosa LAG Esboço do Reconhecimento Ecológico-Agrícola de Moçambique. Lourenço Marques Mem Trab CICA 1955 23 67 226

[B19] Wild H Barbosa LAG Vegetation Map of the Flora Zambesiaca Area Flora Zambesiaca Supplement 1968 Salisbury: M. O. Collins (Pvt) Ltd

[B20] Martin GJ Ethnobotany: A Methods Manual 1995 London: Chapman and Hall

[B21] Cotton CM Ethnobotany: Principles and Applications 1996 Chichester: John Wiley and Sons Ltd

[B22] Van Wyk B A Photographic Guide to Wild Flowers of South Africa 2000 Cape Town: Struik Publishers (PTY) Ltd

[B23] Van Wyk P A Photographic Guide to Trees of Southern Africa 2001 Cape Town: Struik Publishers (PTY) Ltd

[B24] Van Wyk P Field Guide to Trees of the Kruger National Park 2001 Cape Town: Struik Publishers (PTY) Ltd

[B25] Van Wyk B Van Wyk P Field Guide to Trees of Southern Africa 2001 Cape Town: Struik Publishers (PTY) Ltd

[B26] African Plants Database http://www.ville-ge.ch/musinfo/bd/cjb/africa/

[B27] Tropicos.org. Missouri Botanical Garden http://www.tropicos.org

[B28] The International Plant Names Index http://www.ipni.org

[B29] Simbo D An ethnobotanical survey of medicinal plants in Babungo, Northwest Region, Cameroon J Ethnobiol Ethnomed 2010 6 8 10.1186/1746-4269-6-8 20156356 PMC2843657

[B30] Mesfin F Demissew S Teklehaymanot T An ethnobotanical study of medicinal plants in Wonago Woreda, SNNPR, Ethiopia J Ethnobiol Ethnomed 2009 5 28 10.1186/1746-4269-5-28 19821994 PMC2769162

[B31] Bekalo TH Woodmatas SD Woldemariam ZA An ethnobotanical study of medicinal plants used by local people in the lowlands of Konta Special Woreda, southern nations, nationalities and peoples regional state, Ethiopia J Ethnobiol Ethnomed 2009 5 26 10.1186/1746-4269-5-26 19775482 PMC2764637

[B32] Giday M Asfaw Z Woldu Z Teklehaymanot T Medicinal plant knowledge of the Bench ethnic group of Ethiopia: an ethnobotanical investigation J Ethnobiol Ethnomed 2009 5 34 10.1186/1746-4269-5-34 19912633 PMC2780996

[B33] Panghal M Arya V Yadav S Kumar S Yadav P Indigenous knowledge of medicinal plants used by Saperas community of Khetawas, Jhajjar District, Haryana, India J Ethnobiol Ethnomed 2010 6 4 10.1186/1746-4269-6-4 20109179 PMC2826346

[B34] Long C Li S Long B Shi1 Y Liu5 B Medicinal plants used by the Yi ethnic group: a case study in central Yunnan J Ethnobiol Ethnomed 2009 5 13 10.1186/1746-4269-5-13 19389251 PMC2679000

[B35] Bussmann RW Sharon D Traditional medicinal plant use in Northern Peru: tracking two thousand years of healing culture J Ethnobiol Ethnomed 2006 2 47 10.1186/1746-4269-2-47 17090303 PMC1637095

[B36] Asres K Bucar F Kartnig T Witvrouw M Pannecouque C De Clercq E Antiviral Activity Against Human Immunodeficiency Virus Type 1 (HIV-1) and Type 2 (HIV-2) of Ethnobotanically Selected Ethiopian Medicinal Plants Phytother Res 2001 15 62 69 10.1002/1099-1573(200102)15:1<62::AID-PTR956>3.0.CO;2-X 11180526

[B37] Gathirwa JW Rukunga GM Njagi EN Omar SA Mwitari PG Guantai AN Tolo FM Kimani CW Muthaura CN Kirira PG Ndunda TN Amalemba G Mungai GM Ndiege IO The in vitro anti-plasmodial and in vivo anti-malarial efficacy of combinations of some medicinal plants used traditionally for treatment of malaria by the Meru community in Kenya J Ethnopharmacol 2008 115 223 231 10.1016/j.jep.2007.09.021 18065175

[B38] Taylor JSL Elgorashi EE Maes A Investigating the safety of plants used in South African traditional medicine: testing for genotoxicity in the micronucleus and alkaline content assays Environ Mol Mutagenesis 2003 42 144 154 10.1002/em.10184 14556222

[B39] Innocent E Moshi MJ Masimbam PJ Mbwambo ZH Kapingu MC Kamuhabwa A Screening of raditionally used Plants for *in vivo *antimalarial activity in mice African J Trad Complem Altern 2009 6 163 167 10.4314/ajtcam.v6i2.57088 PMC2816569 20209008

[B40] Chinemanaa F Drummondb RB Mavib S De Zoysac I Indigenous plant remedies in Zimbabwe J Ethnopharmacol 1985 14 159 172 10.1016/0378-8741(85)90084-4 4094463

[B41] Johns T Faubert GM Kokwaro JO Mahunnah RLA Kimanani EK Anti-giardial activity of gastrointestinal remedies of the Luo of East Africa J Ethnopharmacol 1995 46 17 23 10.1016/0378-8741(95)01224-2 7475119

[B42] Galvez J Zarzuelo A Crespo ME Utrilla MP Jiménez J Spiessens C de Witte P Antidiarrhoeic activity of Sclerocarya birrea bark extract and its active tannin constituent in rats Phytother Res 2006 5 276 278 10.1002/ptr.2650050611

[B43] Ruffo CK A Survey of medicinal plants in Tabora region, Tanzania Traditional Medicinal Plants 1991 Dar Es Salaam: University Press, Ministry of Health

[B44] Mathabe MC Nikolova RV Lall N Nyazema NZ Antibacterial activities of medicinal plants used for the treatment of diarrhoea in Limpopo Province, South Africa J Ethnopharmacol 2006 105 286 293 10.1016/j.jep.2006.01.029 16545928

[B45] Samie A Obi CL Bessong PO Namrita L Activity profiles of fourteen selected medicinal plants from Rural Venda communities in South Africa against fifteen clinical bacterial species African J Biotechnol 2005 4 1443 1451

[B46] Kokwaro JO Medicinal Plant of East Africa 1976 Kampala, Nairobi, Dar es Salaam: East African Literature Bureau

[B47] Jain A Katewa SS Galav PK Sharma P Medicinal plant diversity of Sitamata wildlife sanctuary, Rajasthan, India J Ethnopharmacol 2005 102 143 157 10.1016/j.jep.2005.05.047 16154303

[B48] Mmatli EE Malerød H Wilson SR Abegaz B Greibrokk T Lundanes E Malterud KE Petersen D Rise F Identification of major metal complexing compounds in Blepharis aspera Anal Chim Acta 2007 597 24 31 10.1016/j.aca.2007.06.042 17658309

[B49] Nanyingi MO Mbaria JM Lanyasunya AL Wagate CG Koros KB Kaburia HF Munenge RW Ogara WO Ethnopharmacological survey of Samburu district, Kenya J Ethnobiol Ethnomed 2008 4 14 10.1186/1746-4269-4-14 18498665 PMC2412850

